# Implementation and Effectiveness of a Pharmacotherapeutic Follow-Up Service for People with Tuberculosis in Primary Healthcare

**DOI:** 10.3390/ijerph192114552

**Published:** 2022-11-06

**Authors:** Célio Rezende Lara-Júnior, Ana Emília de Oliveira Ahouagi, Isabela Vaz Leite Pinto, Debora Gontijo Braga, Thiago Rabelo Andrade, Djenane Ramalho-de-Oliveira, Mariana Martins Gonzaga do Nascimento

**Affiliations:** 1 Center for Pharmaceutical Care Studies, College of Pharmacy, Federal University of Minas Gerais, Belo Horizonte 31270-901, MG, Brazil; 2 Belo Horizonte Municipality, Belo Horizonte 30130-003, MG, Brazil; 3 Pharmaceutical Products Department, College of Pharmacy, Federal University of Minas Gerais, Belo Horizonte 31270-901, MG, Brazil

**Keywords:** tuberculosis, primary healthcare, pharmaceutical services, evidence-based pharmacy practice

## Abstract

Tuberculosis (TB) is a disease of great relevance, responsible for 1.5 million deaths worldwide. Therefore, actions to control TB are necessary, and pharmacists may play an important role, especially in primary healthcare (PHC), where the diagnosis and management of this infection occurs. In a large Brazilian city, pharmacotherapeutic follow-up in PHC has been offered by pharmacists to people with TB since 2018. The objective of this study was to evaluate the implementation and effectiveness of this service though a longitudinal type 1 effectiveness–implementation hybrid study. Data were collected from January 2018 to February 2020 in the pharmaceutical services system. The service indicators were described and effectiveness was evaluated using Poisson regression analysis to compare the incidence of cure among patients using and not using the service. The service was performed in 148 PHC units by 82 pharmacists. Of the total of 1076 treatments, 721 were followed up by pharmacists, and TB was cured more frequently in these cases (90.4% attended vs. 73.5% unattended). The adjusted hazard ratio of cure among patients enrolled in the pharmacotherapeutic follow-up service was 2.71 (2.04–3.61; *p* < 0.001). Pharmacotherapeutic follow-up for people with TB significantly increased the incidence of cure and should be encouraged.

## 1. Introduction

Tuberculosis (TB) has historically been an infectious disease of great concern worldwide. The latest report of the World Health Organization (WHO) reinforced this notion, showing that TB is still highly relevant, although access to treatment has significantly increased in the world. Therefore, it is still necessary to expand access to TB treatment and promote adequate adherence to cure TB, especially because the incidence of *Mycobacterium tuberculosis* infections with increasingly complex resistance profiles has increased worldwide. Thus, the WHO’s report points to the need to treat more than one million people with resistant bacteria; offer preventive actions; and increase investment in the prevention, diagnosis, treatment, and care of TB patients [1].

In the spectrum of developing countries, such as Brazil, the challenges brought by TB are also marked by the difficulties imposed by the precarious urbanization process and sharp social disparities. Brazil ranks 20th in the world regarding the burden of the disease and is also a member of BRICS (a bloc formed by Brazil, Russia, India, China, and South Africa). This group of countries accounted for about 50% of all incident cases of TB in the world and mobilized more than 53% of available resources for disease control actions through domestic sources of funding in 2019 [1,2]. The increase in TB incidence rates in Brazil in recent years, combined with the growing representativeness of populations vulnerable to TB, demonstrates that strategies to control TB are still needed [2].

In this national scenario, primary healthcare (PHC) fulfills the organizational principle of decentralizing the Brazilian public health system and strengthens the bond between community-dwelling TB patients and the healthcare system [3]. Additionally, in the PHC setting, the role of pharmacists to promote the judicious use of medicines has the potential to improve TB treatment initiation and adherence, mainly because it is a complex treatment that involves the use of many medications over a long period [4,5,6].

In the Brazilian city of Belo Horizonte, the pharmacotherapeutic follow-up (PFU) service offered by pharmacists to TB patients was implemented in 2018 in the PHC. Services such as PFU are accessible and easy to reproduce. So far, Belo Horizonte is the Brazilian capital with the second lowest TB incidence rate in the country, but it is still important to assess the impact of the PFU service on these numbers, as well as on the TB cure rates [2]. Some studies have already demonstrated the potential benefit of care provided by pharmacists to TB patients, but there is still a need to explore the impact of their work, especially in “real world” studies [4,7,8,9]. Therefore, the objective of this study was to assess the implementation and effectiveness of a PFU service for people with TB in the city of Belo Horizonte (Brazil).

## 2. Materials and Methods

### 2.1. Study Design

This was a type 1 effectiveness–implementation hybrid study, according to methods encouraged by the WHO for the implementation of research in the healthcare area [6,10]. The study was approved by the Federal University of Minas Gerais (UFMG) Research Ethics Committee (CAAE-25780314.4.0000.5149) and drafted according to the Standards for Reporting Implementation Studies (StaRI) statement [11].

### 2.2. Study Location and Healthcare System

This study was conducted in the public PHC of the municipality of Belo Horizonte, the 6th largest capital city in Brazil, with more than 2.5 million residents. PHC services are the basis of the Brazilian public healthcare system, which is called Unified Health System (Sistema Único de Saúde—SUS). Any person in Brazil (born, living, or visiting the country) may have access to the SUS free of charge, which includes PHC services.

PHC is provided by the SUS in all 9 health regions of Belo Horizonte in 152 PHC centers. In Belo Horizonte, people with TB are preferably managed in the PHC. However, patients with severe TB or people co-infected with HIV are managed in outpatient clinics of public hospitals that specialize in infection management.

Pharmaceutical services in Belo Horizonte are marked by a history of continuous improvements, which has led to the insertion of pharmacists in PHC centers [12]. A total of 82 pharmacists provide pharmaceutical services in PHC, including drug dispensing, medication reconciliation, pharmacotherapy review, health education, and PFU [13].

### 2.3. Pharmacotherapy Follow-Up Service for People with Tuberculosis

Focusing on people with TB, the activities developed by pharmacists in PHC follow the city guidelines, which established the need for PFU for these patients. This service has been provided since 2018 through scheduled appointments for at least three consultations. Ideally, at least one consultation occurs during the intensive phase (until the 15th day after the beginning of treatment, when the patient is usually taking four different drugs); and at least two other consultations occur during the continuation phase (use of two different drugs)—the first of these occurs during the period of change of therapeutic regimens (between the second and third month), and the second occurs at the end of treatment, at six months [13]. TB treatment usually lasts for six months. After treatment, patients are tested to determine if they are cured or need to extend the treatment period. This also leads to additional PFU time. Patients who do not get tested after the end of the treatment are considered dropouts. These procedures are based on the *Manual of Recommendations for Tuberculosis Control in Brazil* [2].

During a consultation, the pharmacist evaluates all medications used by the patient, as well as effectiveness and safety parameters. During the PFU, the pharmacist can request exams and prescribe over-the-counter (OTC) medications if needed [13]. 

Each pharmacist is responsible for providing the PFU service in one to three PHC centers. The PFU service is currently offered to most people with TB (over 70% of people diagnosed, which is about 600 individuals per year). However, the service has yet to be expanded to serve 100% of these patients and increase the number of consultations per patient; hence, assessing the implementation of the PFU service is warranted.

### 2.4. Data Source and Collection

All data were collected and assessed retrospectively for the period from January 2018 to February 2020 from the GERAF (Pharmaceutical Services Management System), which is software developed for the SUS of Belo Horizonte to manage the pharmaceutical services provided. Data were entered monthly by pharmacists responsible for providing pharmaceutical services in Belo Horizonte.

### 2.5. Assessment of Implementation of the Pharmacotherapy Follow-Up Service

To assess the implementation of the PFU service for people with TB, the following variables were described: number of pharmacists offering the PFU service; number of PHC centers where the PFU service was provided; number of people with TB seen by pharmacists; number of pharmaceutical consultations with people with TB; and absenteeism in pharmaceutical consultations. These results were presented using descriptive analysis (absolute number, proportions, mean, standard deviation, median, interquartile range, minimum, and maximum).

### 2.6. Assessment of Effectiveness of the Pharmacotherapy Follow-Up Service

To assess the results of the PFU service, the following data of people with TB included in the service were described: treatments that involved the occurrence of at least one adverse drug reaction (ADR); ADRs identified according to their severity (mild or severe, according to the *Manual of Recommendations for Tuberculosis Control in Brazil*) [2]; and pharmaceutical prescription orders to manage ADRs related to TB treatment. In addition, the following characteristics of people with TB, included or not in the PFU service, were described: sex, age, skin color (black, brown, white, yellow, or native—classification defined by the Brazilian Institute of Geography and Statistics), number of medications used apart from TB treatment, dropout risk stratification (low or high risk), and clinical risk stratification (low, medium, high, and very high risk) according to the tool used in the Belo Horizonte Municipal Health Secretariat [13], and whether the patient with TB was followed by other healthcare workers in Directly Observer Therapy (DOT).

A descriptive analysis of the data was performed by determining the absolute and relative frequencies of the qualitative variables, as well as the mean and standard deviation of the quantitative variables. Differences in the distribution of variables in the exposed (patients included in the PFU service) and unexposed (patients not included in the PFU service) groups were evaluated using: Pearson’s chi-square test in the case of categorical variables; t-test for continuous parametric variables; or Mann–Whitney test for continuous nonparametric variables. The normality of the distribution of the variables was evaluated using the Shapiro–Wilk test.

To assess the effectiveness of the PHU service, the association between the exposure of interest (inclusion in the PFU service—yes vs. no) and the event “TB cure” (yes vs. no) was tested by Poisson regression analysis, which provided estimates of the Hazard Ratio (HR) with a 95% confidence interval (95%CI). Cure was determined by two negative bacilloscopies after six months of treatment or a longer treatment period if necessary, following the *Manual of Recommendations for Tuberculosis Control in Brazil* [2]. The association analysis was presented in an unadjusted model format and in a format adjusted by patients’ characteristics that showed a statistically significant difference between the exposed and non-exposed groups. A statistical significance level of 5% was defined for all analyses. All variables were organized and analyzed in the statistical package Stata^®^ version 12.

## 3. Results

### 3.1. Assessment of Effectiveness of the Pharmacotherapy Follow-Up Service

In 2018 and 2019, there were no cases of tuberculosis in 3 of the 152 PHC centers. The PFU service for patients with TB was offered by all 82 pharmacists in all other PHC centers. A total of 721 patients with TB were included in the PFU service (67.0%), and 355 TB patients were not (33.0%). The total number of pharmaceutical consultations performed was 1709, and the absenteeism rate was 18.4% (315 scheduled consultations missed by patients). The mean number of consultations performed per patient was 2.37 ± 1.38 (minimum = 1; maximum = 9).

### 3.2. Assessment of Effectiveness of the Pharmacotherapy Follow-Up Service

Among patients with TB included in the PFU service, the majority consisted of male patients (64.1%) with brown skin (52.1%). A median age of 45 years was identified, with a higher frequency in the range from 45 to 54 years (minimum = 18; maximum = 89) (Table 1). Most patients had a high risk of dropout (51.2%), low clinical risk (84.3%), and underwent DOT (55.0%) (Table 1).

The median number of drugs used by patients, besides the antituberculosis drugs, was 2; however, this ranged from patients who did not use any other medications during treatment to patients who used up to 21 medications in addition to antituberculosis drugs. In 58.7% of treatments, patients did not present any ADR to the antituberculosis medication, and a pharmaceutical prescription was only needed for 34 cases (Table 1).

The cure rate was higher among patients included in the PFU service (90.4%) than among patients who were not included (73.5%). This difference was statistically significant, and the incidence of cure was about 2.7 times higher for patients included in the service (HR = 2.71; 95%CI = 2.04–3.61) (Table 2), even after adjusting for the number of medications used, which was the only variable that showed a statistically significant difference between the two groups.

## 4. Discussion

The present study demonstrated the implementation and effectiveness of a PFU service offered to people with TB in PHC. To the authors’ knowledge, this is the first study that addresses the evaluation of clinical pharmaceutical services aimed at people with TB using an effectiveness–implementation hybrid study. The adoption of this type of study is encouraged by the WHO, as it is important to evaluate health practices in the real world, not only through the direct description of the activities developed but also through the evaluation of their effectiveness in a more robust way [10,14]. Often, implementation studies assume a direct relationship between process indicators and clinical outcomes, with the effectiveness of the services being automatically assumed. Therefore, patient-level outcome assessments are often not included in study methodologies [10].

In addition, the present study is also relevant because it addresses a service developed in PHC, which is considered the gateway to universal health services such as the Brazilian public health system, and is where most people with TB are diagnosed and treated [2,3]. It is widely known that TB treatment is one of the greatest challenges in PHC because of the treatment dropout risk, making Brazil one of the five countries with the highest treatment dropout rates in the world [1,2]. However, it is important to point out that more severe cases of TB or patients co-infected with HIV are not managed in the Brazilian PHC. Therefore, no patients were hospitalized or died during the follow-up period.

Controlling infectious diseases is necessary due to the potential for the selection of resistant microorganisms [1,15]). Therefore, the proper treatment of TB must go beyond guaranteeing access and should include actions that promote the judicious use of antimicrobials at the individualized level. Initiatives such as the one described in the present study meet these criteria and put the pharmacist at the forefront as a qualified professional to follow up the use of medication and enhance its positive outcomes [16,17]

According to the city´s guidelines, all patients with TB in PHC should be followed up by a pharmacist [13]. However, despite all of the pharmacists in the city offering the PFU service to people with TB in all PHC centers, it was observed that about 33% of patients were not included in PFU service. Additionally, the guidelines state that at least three consultations should be carried out with each patient [13], but the average number of consultations identified in the present study was lower than the minimum recommended number (2.4 ± 1.4).

Several reasons can justify the considerable number of TB patients who were not followed up by pharmacists and the low average number of consultations. Among them, limitations in pharmacist training, lack of documentation of PFU consultations, patient refusal, and patients being followed up by more than one level of care (ex.: hospital and PHC) must be highlighted [18]. In the context studied, it is important to point out that pharmacists are not yet present in all PHC centers in the municipality for 40 h per week. This fact, along with other non-clinical functions performed by pharmacists, such as inventory control, can limit the availability of pharmacists to offer the PFU service to people with TB [13]. Therefore, an organized schedule that optimizes the available time of pharmacists for clinical activities should be developed in order to reach the largest possible number of people with TB.

The rate of absenteeism in pharmaceutical consultations in the PFU service was considerable (18.4%), which demands the implementation of initiatives to reduce it. Absenteeism is considered a worldwide problem in healthcare, causing an increase in waiting lists and demands for urgent care, wasted resources, and reduced productivity. These consequences compromise access to healthcare and increase its costs, stimulate negative attitudes in healthcare professionals, and cause delays in patient treatment [19,20]. For these reasons alone, the absenteeism rate is an important indicator of the effectiveness of the PFU service. However, absence from the consultation may also indicate treatment abandonment, which is even more important. Forgetfulness, communication failures between the service and user, symptom improvement, scheduling issues during working hours, lack of transportation, and scheduled days of the week are the main reasons for absenteeism [19,20]. Additionally, the lack of recognition of the pharmacist as a clinical professional by society can contribute to the rate of absenteeism [21]. 

For purposes of comparability, no PFU studies were found that report the rate of absenteeism, specifically in pharmaceutical consultations, and these data are unprecedented. On the other hand, Clark et al. [22] assessed the frequency of TB follow-up consultations in two groups (patients seen by a physician and nurse and patients seen by a physician, nurse, and clinical pharmacist) and identified a higher frequency of attendance in consultations when the patient was also seen by a pharmacist (53.6% versus 29.3%; *p* < 0.001). In a similar study [23], these values were 81% and 60%, respectively (*p* = 0.018). In this sense, the absenteeism in the PFU service evaluated in the present study is relevant due to the fact that the pharmacist’s performance has the potential to increase consultation attendance for the management of TB as a whole, highlighting the importance of their insertion in the healthcare team. 

In general, the set of results evaluated in this study corroborates the effective implementation of the PFU service. This implementation also shows room for improvement, especially the need to expand the service to ensure the monitoring of all eligible people. This study also reinforces the relevance of the measured indicators and the need to include these indicators in the institutional and individual strategic planning of pharmacists in order to establish the PFU service aimed at people with TB. However, such limitations are minimized from the perspective of being a recently implemented service.

The clinical services provided by pharmacists contribute to the maintenance and recovery of health, as well as the prevention, diagnosis, and treatment of diseases and disorders [24]. In the present study, it was observed that approximately 67% of patients who underwent treatment in PHC in Belo Horizonte were followed up by pharmacists, and this increased the incidence of TB cures by more than 2.71 times. These results are unprecedented, and, to our knowledge, no similar study in the literature has evaluated the effectiveness of a PFU service aimed at people with TB or evaluated any clinical pharmaceutical service for this specific population from the perspective of PHC.

In Brazil, two studies described the results of PFU services in secondary healthcare based on the Pharmaceutical Care Practice offered to 81 and 62 people with TB [7,25]. Resende et al. [7] found that 80% of patients had at least one drug therapy problem (DTP) identified in their pharmacotherapy, while Lopes et al. [25] pointed to a total of 128 identified DTPs, of which 77% were resolved. In contrast to the present study, the impact of the services offered on clinical outcomes was not evaluated, although the resolution of DTPs has the potential to improve health outcomes [7,25].

Other international studies have evaluated the effectiveness of a health education service provided by pharmacists for people with TB. A hospital in Istanbul evaluated the effect of this type of service on adherence to TB treatment and identified a statistically significant difference in outcomes between patients also educated by the pharmacist and those only educated by the doctor and nurse (80.4% versus 42.3%, respectively—*p* < 0.001) [22]. A study with a similar methodology was carried out in a hospital in China, and greater adherence was also identified among people with TB educated by pharmacists (80% versus 50%; *p* = 0.002) [23]. A higher cure rate was also identified among patients educated by pharmacists (71% versus 54%) in the Chinese study, but this difference was not statistically significant (*p* = 0.137) [23].

The cure rate identified with the health education service provided in the hospital in the study that occurred in China was lower than that identified in the present study (90.4%) [23]. This could be expected since PFU is a more complex and longitudinal service, which allows for an individualized and holistic assessment. Furthermore, in the present study, there was a statistically significant difference between the incidence of cures in followed and unattended treatments (cure rate = 73.5%), even after adjusting for variables with uneven distribution between groups (*p* < 0.001).

These results show the effectiveness of the PFU service in increasing an important, if not the most important, outcome in the context of TB. The increase in the cure rate has considerable potential to improve patients’ quality of life, in addition to promoting the control of Koch’s bacillus, as well as reducing the incidence, prevalence, and mortality of TB and the occurrence of resistant strains. From the perspective of the health system, the PFU service can also contribute to reducing costs since the progression of the disease is known to require a greater number of healthcare appointments, including specialized and more expensive services or hospital admission [2].

The present study was limited by the quality of data due to deficient documentation in the pharmaceutical services system. It was observed that despite the existence of a manual with instructions on filling out the Pharmaceutical Services Management System (GERAF) and prior training, the registration of consultations may still be improved with regard to its quality and completeness. However, this limitation was minimized by an analysis of the inconsistencies performed on the data by more than one researcher before its use. Another limitation to be considered is that, despite the high number of patients evaluated in the present study, it cannot be said that the results found have external validity beyond the studied scenario.

Despite these limitations, the results clearly demonstrate the relevance of pharmacists in monitoring people with TB. The PFU service was shown to be viable and potentially effective in terms of promoting the cure of TB in PHC in other municipalities.

## 5. Conclusions

This study presents evidence that the pharmacist may be an important ally for better TB control, contributing to the patient´s cure. Furthermore, describing the pioneering spirit of the municipality of Belo Horizonte in bringing pharmacists to play a leading role in the monitoring of TB patients in PHC serves as a reference for other cities for the adoption of public measures that rely on the pharmacist in patient-centered clinical care.

## Figures and Tables

**Table 1:** Characteristics of patients undergoing tuberculosis treatment in primary healthcare centers, included or not included in the pharmacotherapeutic follow-up (PFU) service of 2018–2020. Belo Horizonte-MG. Brazil.

Variable	Included in the PFU Service	Not Included in the PFU Service	*p*-Value
	(N = 721)	(N = 355)	
**Sex—N (%)**			
Female	258 (35.9)	115 (32.5)	0.265 *
Male	460 (64.1)	239 (67.5)	
**Age—Median** **(interquartile range)**	45 (26)	43 (23)	0.880 **
**Race—N (%)**			
White	207 (30.0)	113 (35.2)	0.076 *
Yellow	2 (0.3)	1 (0.3)	
Brown	360 (52.1)	145 (45.2)	
Black	122 (17.6)	60 (18.7)	
Native	0 (0.0)	2 (0.6)	
**Number of medications used—Median** **(interquartile range)**	2 (5)	1 (4)	0.0001 **
**Dropout risk—N (%) ^#^**			
Low	352 (48.8)	157 (45.0)	0.239 *
High	369 (51.2)	192 (55.0)	
**Clinical risk—N (%) ^#^**			
A	608 (84.3)	302 (86.0)	0.418 *
B	106 (14.7)	48 (13.7)	
C	7 (1.0)	1 (0.3)	
D	0	0	
**Directly Observed Treatment—N (%)**			
No	322 (44.9)	62 (40.0)	0.258 *
Yes	394 (55.1)	93 (60.0)	
**Adverse Drug Reaction to Antituberculosis Drug—N (%)**			
None	439 (60.9)	N/A	N/A
Mild	257 (35.6)	N/A	N/A
Severe	25 (3.5)	N/A	N/A
**Pharmaceutical Prescription—N (%)**			
Yes	31 (4.3)	N/A	N/A
No	690 (95.7)	N/A	N/A

* Pearson’s chi-square; ** Mann–Whitney test *or t*-test; ^#^ Calculated according to the clinical risk and dropout stratification of Belo Horizonte city.

**Table 2:** Comparison of the incidence of cure among people with tuberculosis included or not included in pharmacotherapeutic follow-up (PFU) service of 2018–2020. Belo Horizonte-MG. Brazil.

Cure	Included in PFU Service	Not Included in PFU Service	HR (95%CI; *p*-Value) *	HR (95%CI; *p*-Value) **
	(N = 721)	(N = 355)		
Yes	652 (90.43)	261 (73.52)	2.77 (2.08–3.67; <0.001)	2.71 (2.04–3.61; <0.001)
No	69 (9.57)	94 (26.48)		

* Hazard Ratio from the robust Poisson regression. ** Hazard Ratio from the robust Poisson regression adjusted by number of medications used.

## Data Availability

Restrictions apply to the availability of these data. Data were obtained from the SUS of Belo Horizonte and are available upon request to the Research Nucleus of the City Government of Belo Horizonte.
